# Case–control association study between polygenic risk score and COVID-19 severity in a Russian population using low-pass genome sequencing

**DOI:** 10.1017/S0950268824001778

**Published:** 2024-12-26

**Authors:** Arina Nostaeva, Valentin Shimansky, Svetlana Apalko, Ivan Kuznetsov, Natalya Sushentseva, Oleg Popov, Anna Asinovskaya, Sergei Mosenko, Lennart Karssen, Andrey Sarana, Yurii Aulchenko, Sergey Shcherbak

**Affiliations:** 1 City Hospital No. 40 of Kurortny District, St. Petersburg State Budgetary Healthcare Institution, Sestroretsk, Russia; 2 St. Petersburg State University, St. Petersburg, Russia; 3 Center of Life Sciences, Skolkovo Institute of Science and Technology, Moscow, Russia; 4 PolyKnomics BV, s-Hertogenbosch, The Netherlands

**Keywords:** COVID-19, genetic predisposition, genome-wide association studies, low-pass whole genome sequencing, polygenic risk score

## Abstract

The course of COVID-19 is highly variable, with genetics playing a significant role. Through large-scale genetic association studies, a link between single nucleotide polymorphisms and disease susceptibility and severity was established. However, individual single nucleotide polymorphisms identified thus far have shown modest effects, indicating a polygenic nature of this trait, and individually have limited predictive performance. To address this limitation, we investigated the performance of a polygenic risk score model in the context of COVID-19 severity in a Russian population. A genome-wide polygenic risk score model including information from over a million common single nucleotide polymorphisms was developed using summary statistics from the COVID-19 Host Genetics Initiative consortium. Low-coverage sequencing (5x) was performed for ~1000 participants, and polygenic risk score values were calculated for each individual. A multivariate logistic regression model was used to analyse the association between polygenic risk score and COVID-19 outcomes. We found that individuals in the top 10% of the polygenic risk score distribution had a markedly elevated risk of severe COVID-19, with adjusted odds ratio of 2.9 (95% confidence interval: 1.8–4.6, *p*-value = 4e-06), and more than four times higher risk of mortality from COVID-19 (adjusted odds ratio = 4.3, *p*-value = 2e-05). This study highlights the potential of polygenic risk score as a valuable tool for identifying individuals at increased risk of severe COVID-19 based on their genetic profile.

## Introduction

COVID-19 (coronavirus disease 2019) is a contagious illness caused by the severe acute respiratory syndrome coronavirus 2 (SARS-CoV-2). The majority of individuals who contract the virus exhibit mild to moderate respiratory symptoms and can recover without requiring specific medical treatment. However, in certain cases, the disease can manifest in a severe form, requiring medical intervention [1, 2].

Apart from external factors such as virus characteristics and the effectiveness of healthcare, certain host-related factors such as increasing age, male gender, and pre-existing chronic diseases such as hypertension, cardiovascular disease, or diabetes have been associated with susceptibility and severity of COVID-19 [3, 4]. However, these risk factors alone cannot fully explain the wide variation observed in the disease severity, which can range from asymptomatic cases to acute respiratory distress syndrome and even death [5–7].

To gain insights into the aetiology of COVID-19, large-scale genetic association studies including both rare and common genetic variants have employed various study designs. These investigations, along with subsequent follow-up studies, have expanded our understanding of the disease and provided potential avenues for its treatment. The COVID-19 Host Genetics Initiative (HGI) was established to investigate the role of human genetics in the severity and susceptibility of COVID-19 [8]. This global effort aims to conduct a meta-analysis of multiple COVID-19 genome-wide association studies (GWAS) and to identify single nucleotide polymorphisms (SNPs) associated with SARS-CoV-2 infection or severe manifestations of COVID-19. Combining genetic data from up to 49562 cases and 2 million controls across 19 countries through case–control meta-analyses, these studies have implicated a total of 13 independent genome-wide significant loci associated with COVID-19-related traits [9]. Several of these loci represent potentially actionable mechanisms in response to infection, such as innate antiviral immune signalling, regulation of organ-specific inflammatory responses, and upregulation of cell receptors [10, 11].

The effects of individual genetic variants identified so far are generally small, consistent with the polygenic architecture. An individual who tests negative for a specific genetic risk variant may still have a high genetic risk due to other unmeasured genetic factors. While every single variant only explains a small portion of the risk for severe COVID-19, combining multiple genetic variants into a polygenic risk score (PRS) can offer a better prediction of the risk. PRS allows for the aggregation of the effects of multiple SNPs into a single score, which can be practically applied to individuals within a population [12]. Conventionally, a polygenic score is defined as a weighted linear combination of allele counts for SNPs observed in an individual’s genome [13].

Several studies have explored the development and evaluation of PRS for potential clinical applications, revealing that PRS is associated with an increased risk of severe COVID-19, most of these studied cohorts consisting predominantly of individuals of Western European ancestry [15–17]. Using 1582 SARS-CoV-2 positive participants from the UK Biobank (1018 with severe COVID-19 and 564 without severe COVID-19) and 64 SNPs for PRS calculation, Dite et al. developed and validated a clinical and genetic model for predicting the risk of severe COVID-19. Only 13% of participants from this study were non-European, and the model including PRS alone had an area under the receiver operating characteristic curve (AUC) of 0.68 [17].

While one recent study included African and South Asian groups, the associations with COVID-19 outcomes were limited by applying a PRS based on only six SNPs [18]. Another study that considered non-Western European populations was constrained by its focus on a specific Russian cohort (athletes) and also included only six genetic polymorphisms in the PRS assessment [19]. The multi-ethnic approach implemented in a recent paper using UK Biobank data allowed the application of PRS, based on 17 SNPs, to diverse populations, where a PRS model showed meaningful prediction performance across Black and Asian cohorts [20, 21].

While the study’s findings suggest that PRS may have the potential as a predictive marker for COVID-19 severity and support their potential use in risk stratification and personalized healthcare approaches, the limitations of these studies underscore the need for further research to enhance the predictive capability of PRS in the context of COVID-19.

Our study aimed to investigate the performance of the PRS model in the Russian population. The genomes of study participants (319 individuals with severe COVID-19 and 663 with moderate or without disease) were assessed using low-coverage (with a mean depth of 5x) sequencing. Next, we developed a genome-wide PRS model for COVID-19 severity using the summary statistics from the HGI consortium. We demonstrated that PRS, incorporating information from more than a million common genetic variants, can identify individuals with markedly elevated risk of severe COVID-19 course: adjusted OR = 2.9 (95% confidence interval (CI): 1.8–4.6, *p*-value = 4e-06) for individuals in the top 10% of the PRS distribution, and can moderately improve the quality of prediction (AUC increased from 0.68 to 0.71) compared to a model including only demographic and clinical information.

## Materials and methods

### Study participants and genetic sequencing

As part of the COVID-19 study, biomaterial (blood) and clinical data from COVID-19 patients hospitalized in the infectious disease department of the St. Petersburg State Budgetary Healthcare Institution ‘City Hospital No. 40 of Kurortny District’ were collected. For each participant, retrospective and prospective access to electronic health data was available, including the Hospital Information System (HIS).

In this work, low-coverage sequencing with an average coverage of 5x was performed for 1101 individuals divided into 41 batches. Low-coverage sequencing, also called low-pass whole genome sequencing (LP-WGS), is a low-cost, high-throughput DNA sequencing technology used to accurately detect genetic variation in the genomes of multiple species [22]. LP-WGS is the type of WGS with genome coverage from 0.5x to 5x [23, 24]. Using imputation algorithms, this technology provides high variant detection accuracy with low sequence coverage. LP-WGS and subsequent imputation yields, across the genome, more accurate genotypes than imputation using array-based genotyping data, allowing for increased power in GWAS studies and more accurate results in polygenic risk prediction [25].

Before sequencing, a preliminary analysis and quality control of the data were performed. This was followed by a preprocessing stage that used blocking and randomization techniques to design sequencing batches to reduce potential biases and ensure an even distribution of sample characteristics, such as age, sex, and case/control status.

Genomic DNA isolation was performed with QIAcube, using a QIAamp DNA Blood Mini Kit. DNA concentration was measured with a Promega QuantiFluor dsDNA System. Library preparation was done using an MGIEasy FS DNA Library Prep Set. Sequencing was done on an MGISEQ-2000 sequencing machine with the DNBSEQ-G400RS high-throughput sequencing set (FCL PE150, 540 G).

### Variant calling, imputation, and quality control

Quality control (FastQC, version 0.11.9) [26], alignment (BWA, version 0.7.17) [27], deduplication (samtools, version 1.16.1), and variant calling (bcftools, version 1.16) were performed for the reads obtained from sequencing [28]. Imputation of the resulting data was then performed using the GLIMPSE tool (version 1.1.1) [29], which allowed the imputation of low-coverage sequencing data. To improve the imputation quality, only bi-allelic sites were retained from the LP-WGS BAM data and processed with bcftools. Then iterative refinement of the genotype likelihood using the reference panels with a segmentation size of 2 Mb with a buffer size of 200 kb produced imputed dosages, and multiple chunks within each chromosome were ligated. A panel of the 1000 Genomes Project with high coverage [30], including high-quality single nucleotide variants and insertion-deletion mutations (SNV- and INDELs) from over 3000 individuals, was used as a reference sample.

Subsequently, variants with an INFO score below 0.7 and a minor allele frequency (MAF) less than 0.1% were excluded from the analysis, following established protocols [9, 13]. After applying filters for variant and individual call rates above 90%, five participants were removed. Genetic sex was inferred based on X chromosome heterozygosity using PLINK’s implementation, where individuals with heterozygosity estimates (F) less than 0.2 were classified as female and those with F greater than 0.8 were classified as male. A total of 24 participants with discordant genetic sex were excluded. First- and second-degree relatives were identified using the KING-robust method [31] and removed from the analysis, with a kinship threshold of 0.125. This step resulted in the exclusion of 32 participants (one member of each pair). The analysis was then restricted to a list of HapMap3 variants [32] included in the PRS models. All genotype extraction and quality control procedures were performed using PLINK 1.90 software [33].

### Establishing COVID-19 severity

The criteria for a mild course of COVID-19 were defined as having a body temperature below 38°C, cough, weakness, sore throat, and the absence of indicators characteristic of a moderate or severe course. A moderate course of COVID-19 was characterized by fever with a temperature above 38°C, a respiratory rate greater than 22 breaths per minute, shortness of breath, pneumonia as detected by computed tomography (CT) scan of the lungs, and oxygen saturation (SpO_2_) less than 95%. Severe disease was clinically and radiologically defined by a respiratory rate exceeding 30 breaths per minute, SpO_2_ < 93%, PaO_2_/FiO_2_ < 300 mm Hg, progression of changes in the lungs characteristic of COVID-19, and pneumonia as indicated by CT data, including an increase in the prevalence of identified changes by more than 25%.

Additional criteria for severe disease included the appearance of signs of other pathological conditions, changes in the level of consciousness, unstable haemodynamics (systolic blood pressure less than 90 mm Hg or diastolic blood pressure less than 60 mm Hg, and urine output less than 20 ml/h), and a quick Sequential Organ Failure Assessment (qSOFA) score > 2 points. Lastly, the criteria for an extremely severe course included signs of acute respiratory distress syndrome (ARDS) requiring respiratory support (invasive ventilation), septic shock, and multiple organ failure.

### Ascertainment of covariates

The covariates of the present study were as follows: age, sex, and comorbidities, including myocardial infarction (MI), congestive heart failure (CHF), peripheral artery disease (PAD), cerebrovascular disease (CD), chronic obstructive pulmonary disease (COPD), diabetes, and kidney damage. The information pertaining to the comorbidities was derived from the electronic medical records. The first ten principal genetic components (PCs) were also included as covariates to adjust for population genetic structures and avoid bias, as per current recommendations [13]. The computation of PCs was done by PLINK 1.90 software, via the – pca option [33]. In addition to genetic quality control measures, 58 participants were excluded from the analysis because information on their clinical parameters was missing and could not be obtained. As a result, the rate of missing values for any comorbidity is 0%.

### Construction of PRS models

The calculation of PRS values relies on both genotype data from the target individuals and a PRS model. To derive a PRS model, GWAS are used to estimate the effect sizes of SNPs [34]. However, a GWAS gives the marginal effect size for each SNP estimated by a regression model that ignores linkage disequilibrium (LD) structure. As a result, to construct a PRS model that incorporates multiple SNPs, the SNP effects must be re-estimated while accounting for LD structure.

We used the GWAS from the COVID-19 HGI consortium (release 7). These results were obtained by the meta-analysis that combined the results of 60 individual studies from 25 countries, with a total of 18000 severe cases of COVID-19 and more than a million controls who either did not have a severe disease course or were not affected by COVID-19 during the study period.

To re-weight the effect sizes, we used SBayesR (version gctb_2.02), a powerful Bayesian tool that uses summary statistics from GWAS [14]. This tool re-weights the effects of each genetic variant based on the marginal estimate of its effect size, statistical strength of association, the degree of correlation between the variant and other variants nearby, and tuning parameters. We used the default values for tuning parameters. It also requires a GCTB-compatible LD matrix file based on individual-level data from a reference population, and for this analysis, we used a shrunk sparse GCTB LD matrix from 50000 individuals of European ancestry in the UK Biobank dataset [35].

PRS values were calculated as a weighted sum of allele counts:

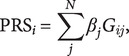

where 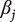 is the re-weighted effect size of the 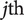 SNP, 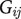 is the genotype of the 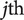 SNP for 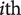 individual, and *N* is the number of SNPs from the model. PLINK 1.90 software [33] was used for PRS calculation (via the – score option).

### Statistical analysis and association testing

Logistic regression of COVID-19 outcomes on PRS was then conducted using R (version 4.3.2) [36] and Python (version 3.8.17) [37], adjusted for covariates (sex, age, comorbidities, and the first ten PCs). The discriminative power of models in identifying high-risk individuals was then assessed using receiver operating curve (ROC) analysis. The area under the ROC (AUC) was calculated for full models (consisting of covariates and PRS) and base models (covariates-only). Because of limited sample size, we used the stratified k-fold cross-validator (*k* = 5) to estimate a mean AUC for each logistic regression model. The confidence interval for the AUC was calculated using the formula given by Hanley and McNeil [38]. Increment in AUC (ΔAUC) was reported based on the difference between the two models, reported as the discriminative or predictive power conferred by PRS. The bootstrap procedure for differences between classifiers was used to calculate the significance (*p*-value) of an increment in AUC.

Once the PRS was calculated, individuals were separately stratified into quintiles for PRS. Next, they were categorized into low genetic risk (decile 1, bottom 10% of cohort), intermediate risk (decile 2–9, middle 80%), and high risk (decile 10, top 10%). For each group, we applied the non-parametric Kaplan–Meier estimator [39] to estimate the cumulative density curve and the log-rank test (with other groups used as a reference), which is the statistical test for comparing the survival distributions of two or more groups. Participants’ birthdates were considered the starting point, and their respective enrolment dates in the study served as the endpoint. For this analysis, COVID-19 outcomes (severe cases or death due to COVID-19) were defined as events, while all other observations were treated as censored data. We performed a multivariate Cox regression analysis for the whole cohort using PRS values, sex, comorbidities, and the first ten principal components of genetic variation as parameters. To compare survival with changes in PRS, we plotted the partial effects of PRS on outcome, where we fixed the values of the covariates and used the mean PRS values in each of the three groups described above. The survival analysis was performed using Python (version 3.8.17) [37].

## Results

### Participant characteristics

The participants of the study were the patients of the Infectious Disease Department of the St. Petersburg State Health Care Institution ‘City Hospital No. 40, Kurortny District’ who were admitted for treatment with SARS-CoV-2 infection (confirmed by polymerase chain reaction), and healthy individuals. Healthy individuals are defined as people who did not require COVID-19 medical treatment at the time of the study (between April 2020 and March 2022).

Table 1 shows the participants’ characteristics. Of the 982 participants who passed quality control (Supplementary Figure 1), 431 (44%) were female, with a mean age of 60 years, while for 551 (56%) male participants, the mean age was 56 years. Overall, 814 (83%) of all participants had COVID-19, of which 319 (39%) had severe COVID-19. Separation according to the severity of the disease was carried out according to the clinical and radiological data (Methods). The case group included 319 patients (199 men and 120 women, 63 ± 14 years) with the severe and extremely severe course of COVID-19. The control group, which combined mild and moderate forms of COVID-19, as well as healthy individuals, included 663 patients (352 men and 311 women, 56 ± 16 years). Participants’ comorbidity characteristics stratified by COVID-19 severity and mortality are shown in Supplementary Tables 1–7.

**Table 1.** COVID-19-related characteristics of the participants

Characteristics		Male	Female
Mean age (SD)		56 (15)	60 (16)
Healthy individuals		101	67
Patients required treatment by severity (course of COVID–19)	Mild	74	63
	Moderate	177	181
	Severe	198	117
	Extremely severe	1	3
Outcome	Death	86	47
	Recovery or no disease	450	384

### Low-coverage sequencing and imputation

For all participants, low-pass whole genome sequencing (LP-WGS) was performed with a depth of 5x genome coverage. To evaluate the efficiency of LP-WGS in the PRS context, we calculated PRS values for a participant (not included in the study population) sequenced in each of the batches to control the quality of the sequencing process. The coefficient of variation (CV) was equal to 0.5%, demonstrating excellent technical reproducibility.

### Testing associations between PRS and COVID-19 severity

**Figure 1.**
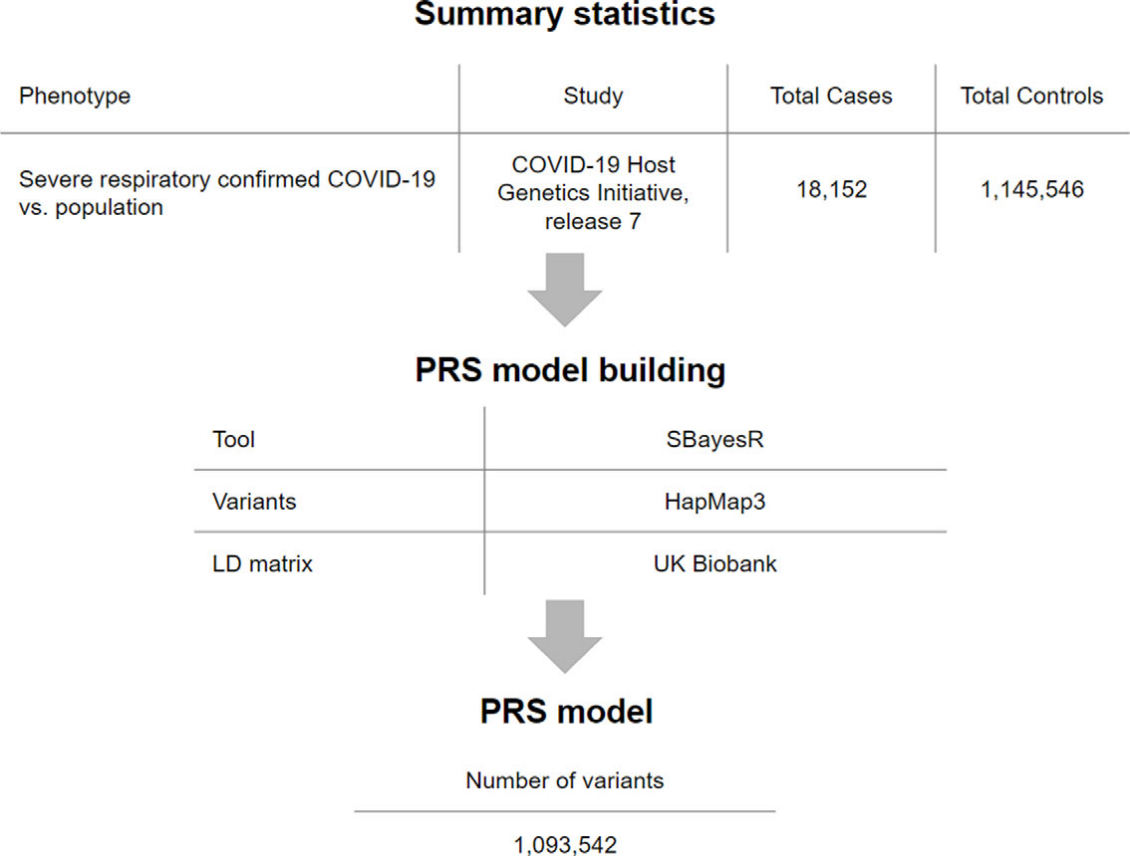
Study design and workflow. The PRS model for COVID-19 severity was derived by combining summary association statistics from the COVID-19 Host Genetics Initiative consortium and a linkage disequilibrium reference panel of 50,000 individuals of European ancestry from the UK Biobank data set. As a computational algorithm, SBayesR was used, which is a Bayesian approach to calculate a posterior mean effect for all variants based on a prior (effect size in the previous GWAS) and subsequent shrinkage based on linkage disequilibrium. The PRS model was restricted by the list of variants from HapMap3 and included about one million variants.

Using the summary-statistics-based approach, leveraging information from a robust meta-analysis, we developed a PRS model, which incorporated 1093542 common genetic variants. Subsequently, we calculated individual PRS values using the obtained PRS model (Figure 1).

**Figure 2.**
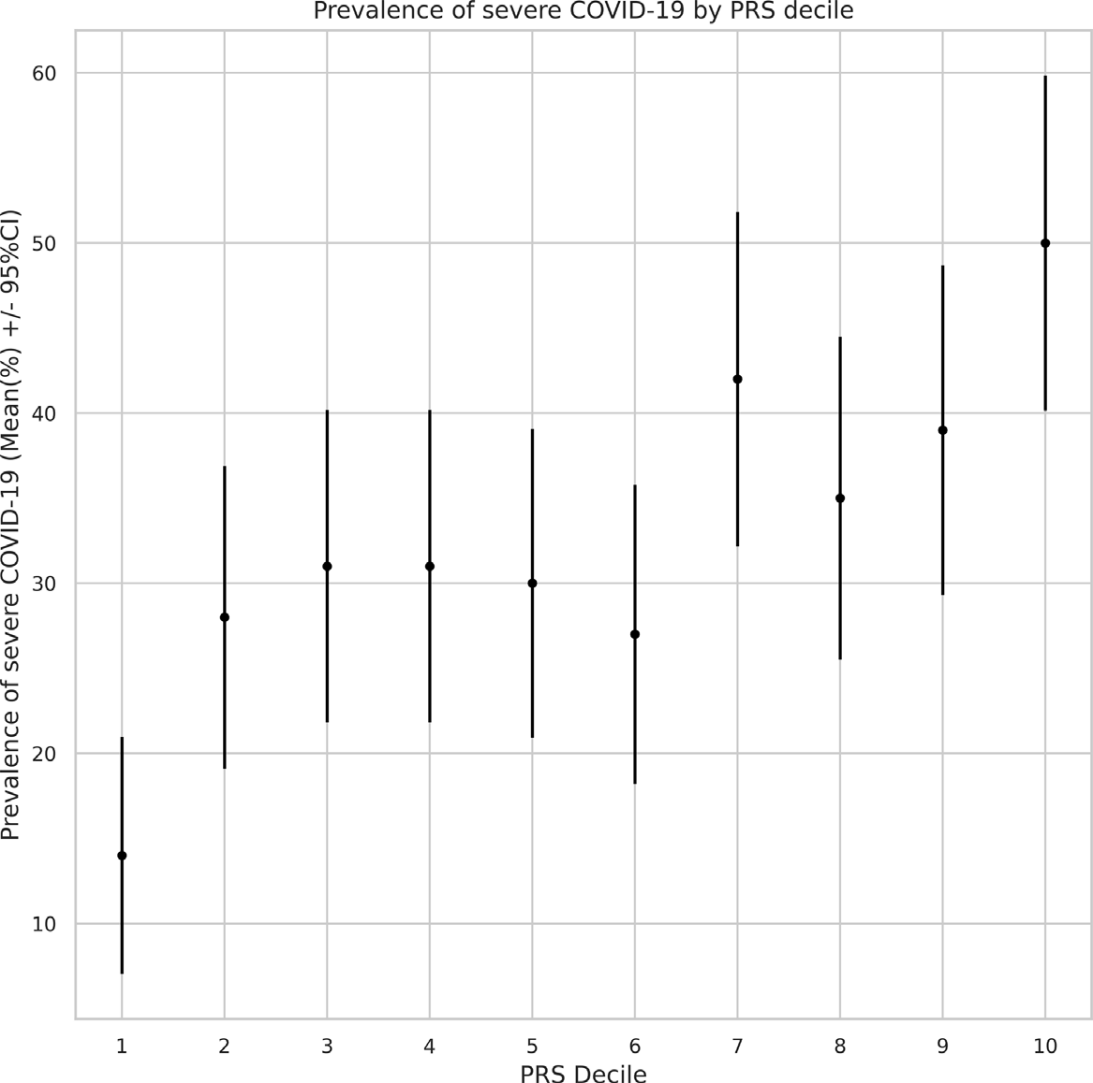
Prevalence of the severe COVID-19 according to PRS decile. All participants (*N* = 982) were stratified by decile of the PRS distribution. The average prevalence in per cent and 95% CI within each decile are displayed.

**Figure 3.**
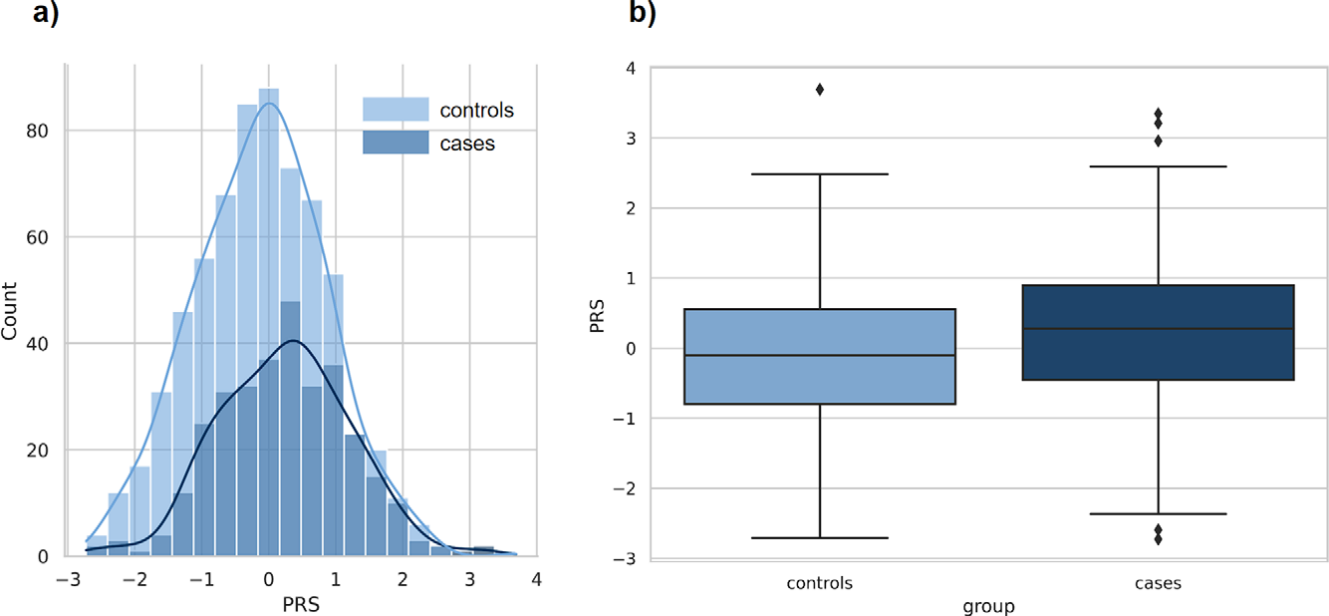
Comparison of distributions of PRS values between the groups with and without severe COVID-19. (a) Distribution of PRS in the groups with (*N*_cases_ = 319) and without (*N*_controls_ = 663) severe COVID-19. The *x*-axis represents PRS, with values scaled to a mean of 0 and a standard deviation of 1 (in the total sample) to facilitate interpretation. (b) PRS values among cases versus controls. Within each box plot, the horizontal lines reflect the median, the top, and bottom of each box reflect the interquartile range, and the whiskers reflect the rest of the distribution, except for points that are determined to be ‘outliers’.

Across the study population, the PRS was normally distributed with the prevalence of severe COVID-19 rising in the right tail of the distribution, from 15% in the lowest decile to around 50% in the highest decile (Figure 2). We compared the distributions of PRS values between severe cases and the control group. Comparison of the mean PRS values, performed using Student’s *t*-test for two independent samples, showed a significant difference (*p*-value = 1e-07), with PRS values larger in severe cases (Figure 3). Next, we found that the 10% of the population with the highest PRS values had an OR = 2.3 for COVID-19 (95% CI: 1.5–3.6, *p*-value = 0.0001).

### Adjusted and ROC analysis

We analysed the association between PRS and severe COVID-19 using a multivariate logistic regression model adjusted for sex, age, comorbidities, and the first ten principal components of genetic variation. In the adjusted model, a highly significant association between PRS and severe COVID-19 was found: OR = 1.54 per standard deviation (95% CI: 1.3–1.8 with *p*-value = 2e-08, Supplementary Table 8). High values of PRS (the 10% of PRS distribution) were associated with the adjusted OR = 2.9 (95% CI: 1.8–4.6, *p*-value = 4e-06, Supplementary Table 9).

**Figure 4.**
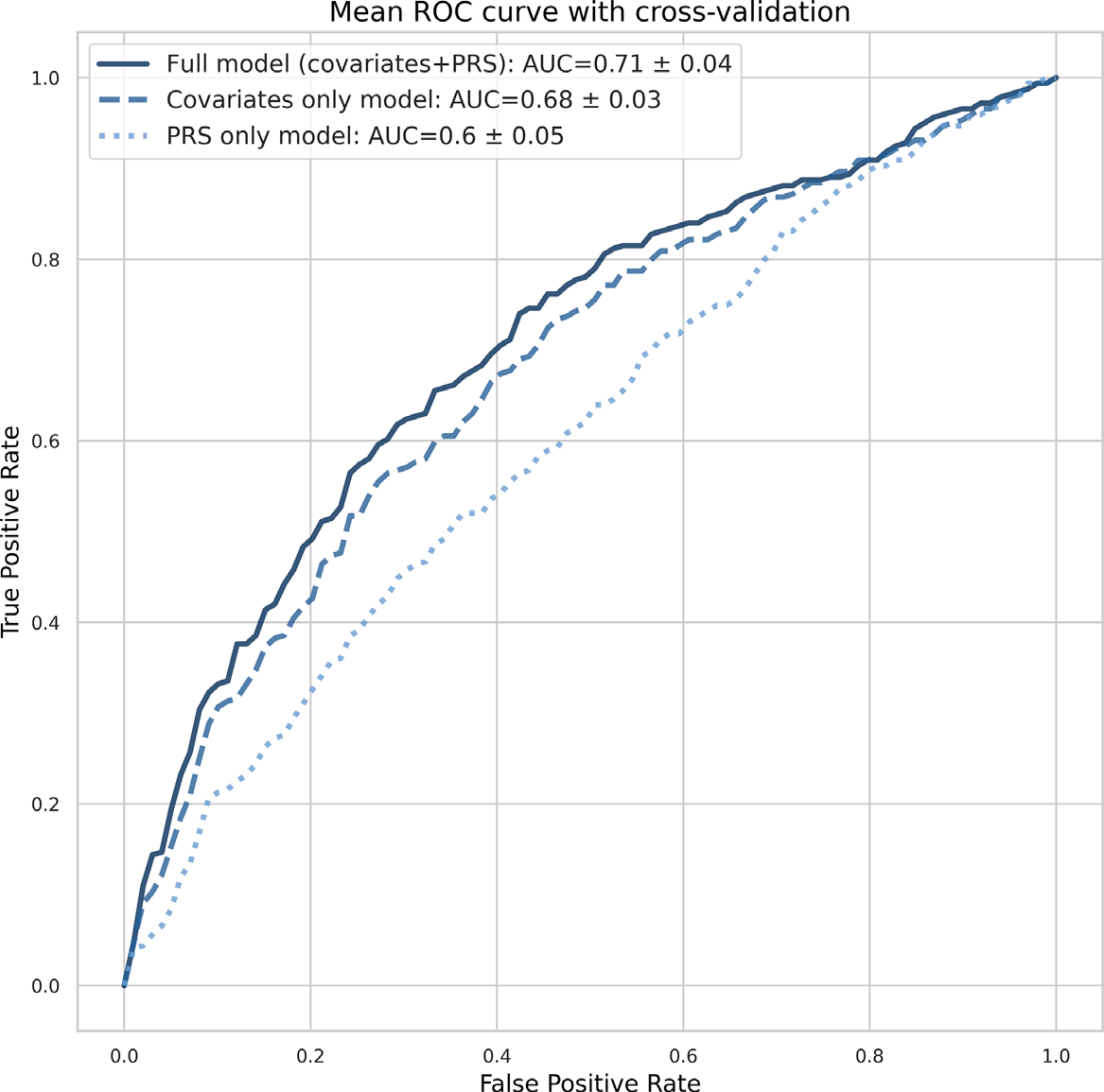
The comparison of receiving operating curves for three logistic regression models. The full model included the covariates (sex, age, comorbidities, and the first ten PCs) and the PRS, while the covariates-only model excluded the PRS.

With the applied cross-validation approach, the analysis showed moderate improvements in AUC with the addition of PRS to the base model containing only covariates (Supplementary Table 10). The model predicting the risk of severe COVID-19 had an AUC of 0.68 (95% CI: 0.6–0.76 by the formula given by Hanley and McNeil [38]) for a model excluding PRS, and it increased up to 0.71 (95% CI: 0.62–0.79) when PRS was included (Figure 4). However, given the limited sample size, this result was insignificant (*p*-value > 0.05 for increment in AUC). The PRS-only model had an AUC of 0.6 (95% CI: 0.52–0.69, Supplementary Table 11).

### Testing associations between PRS and COVID-19 mortality

**Figure 5.**
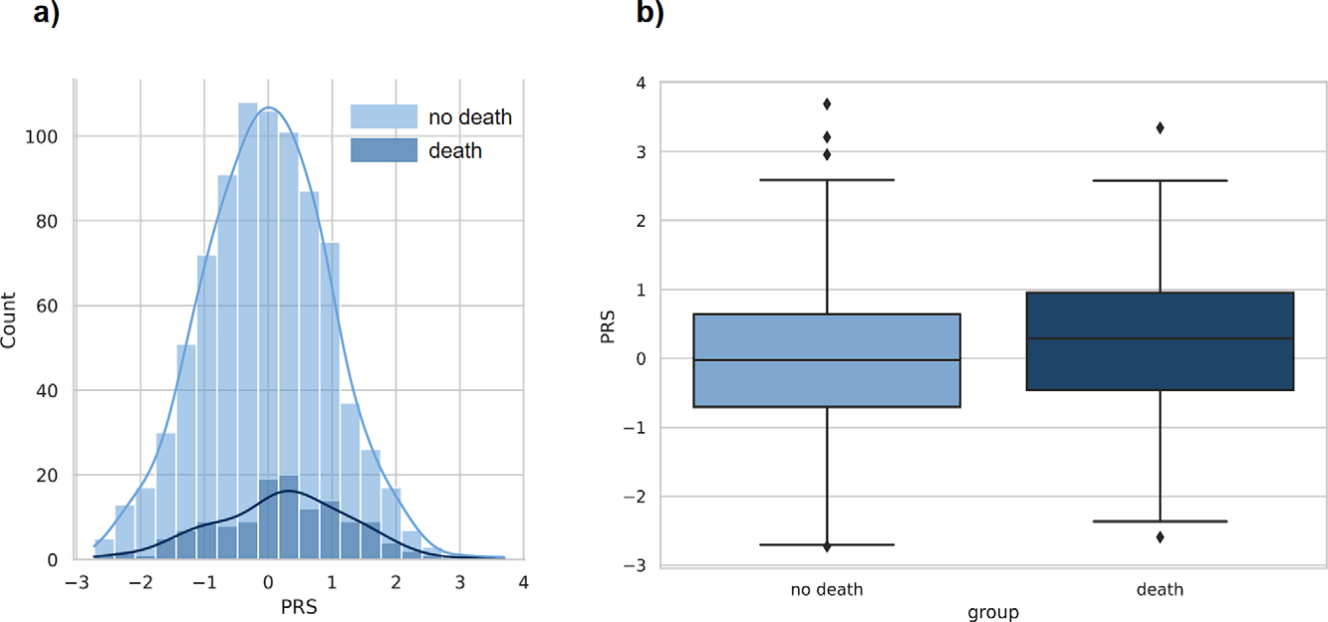
Comparison of distributions of PRS values between the groups with and without death outcome. (a) Distribution of PRS in the groups with (*N*_death_ = 133) and without (*N*_no death_ = 849) death outcome of COVID-19. The *x*-axis represents PRS, with values scaled to a mean of 0 and a standard deviation of 1 (in the total sample) to facilitate interpretation. (b) PRS values among cases versus controls. Within each box plot, the horizontal lines reflect the median, the top, and bottom of each box reflect the interquartile range, and the whiskers reflect the rest of the distribution, except for points that are determined to be ‘outliers’.

The severe form of the disease is associated with an increased risk of death. We compared the mean PRS values for groups with different COVID-19 outcomes (death vs no death or no disease). Results showed a significant difference in mean PRS (*p*-value = 0.006, Figure 5).

Then, to assess how much the risk of death is associated with an increased PRS value, we calculated the odds ratio (OR) for death between the group with the highest PRS values (10%) and others. The resulting OR was 2.0 (95% CI: 1.1–3.4) with *p*-value = 0.01. Thus, in the group with the highest PRS values, the probability of death due to severe disease was almost doubled. In an adjusted model with the same covariates as in the analysis of COVID-19 severity, a significant association between PRS and COVID-19 mortality was found: OR = 1.5 per standard deviation (95% CI: 1.2–1.9 with *p*-value = 0.0007, Supplementary Table 12). High values of PRS (the 10% of PRS distribution) were associated with the adjusted OR = 4.3 (95% CI: 2.2–8.5, *p*-value = 2e-05, Supplementary Table 13).

### PRS-based survival analysis for COVID-19 severity and mortality

**Figure 6.**
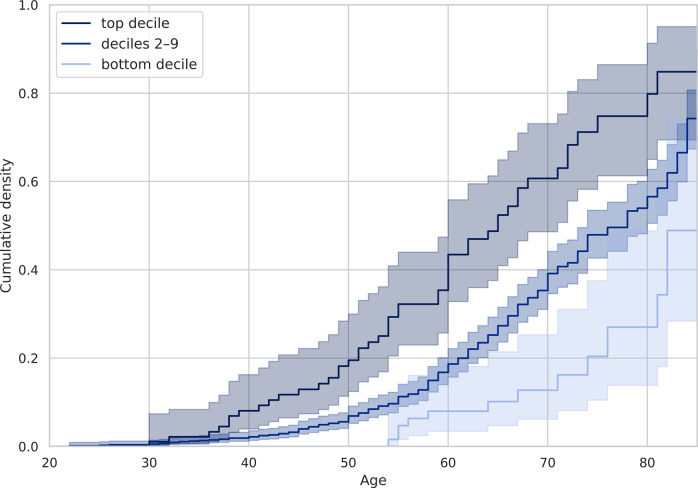
Association of PRS with COVID-19 severity. All participants (*N* = 982) were stratified into three categories, based on their PRS: bottom decile, deciles 2–9, and top decile. The Kaplan–Meier curve is plotted according to the PRS category.

Next, we hypothesized that PRS for severe COVID-19 would be associated with a higher risk of severe COVID-19 in early age. In Kaplan–Meier analyses, which is a non-parametric statistic used to estimate the survival function from lifetime data, we divided the sample into three groups: 10% of all individuals with the highest PRS values, 10% of all individuals with the lowest PRS values, and the rest (Figure 6). The analysis showed that people from the group with high PRS values started to have an increased risk of severe COVID-19 in comparison with other groups already before the age of 40 years (*p*-value < 2e-8 for the log-rank test). For example, the average risk of a severe course, which is reached at the age of 60 years, in the group with the highest PRS is reached before 50 years of age. Adjustment for covariates (sex, comorbidities, and the first ten PCs of genetic variation) using a multivariate Cox regression analysis showed similar results (Supplementary Figure 2) with the hazard ratio (HR) for PRS = 1.39 (*p*-value < 1e-7, Supplementary Table 14). The same analysis for the COVID-19 mortality showed similar results, where people from the group of high PRS values started to have an increased mortality risk of COVID-19 in comparison with other groups already before the age of 50 years (*p*-value < 2e-6 for the log-rank test, Supplementary Figures 3 and 4) with HR for PRS = 1.44 (*p*-value < 1e-3, Supplementary Table 15).

## Discussion

In this study, we constructed a polygenic risk model for predicting the severity and mortality risk of COVID-19 and applied it to a target cohort of 982 Russian participants. Comparing the distributions of a PRS, incorporating information from more than one million common genetic variants, between the case and control groups revealed significant differences, indicating meaningful associations between the PRS and COVID-19 severity and mortality. We also demonstrated the potential of LP-WGS with 5x coverage to be used for PRS calculation followed by genetic predisposition prediction.

Our main objective was to evaluate the predictive ability of PRS for COVID-19 severity in a population of Eastern European ancestry. To achieve this, we developed a logistic regression model that included demographic and clinical covariates and the full model that also incorporated the PRS. Comparison between these models demonstrated that incorporating PRS moderately enhanced the predictive accuracy. These findings align with a previous analysis by Huang et al. [16], where PRS values for severe COVID-19 were constructed using 112 SNPs in 430,582 participants from the UK Biobank study. In that work, AUC was calculated for a model including only covariates and for the full model, which also included the PRS. For the first model, the AUC was 0.789, while in the full mode, the AUC was 0.794 (*p*-value = 0.002 for increment in AUC). The higher overall prediction accuracy of the model could be attributed to the usage of information on additional covariates (hypertension, chronic respiratory infections, asthma, physical activity, smoking status, alcohol consumption, and body mass index). Although our PRS, based on approximately one million SNPs, offered stronger improvement in AUC (0.026 in our study vs 0.005 in Huang et al.), this result was insignificant, given the limited sample size of our study. The higher contribution of the PRS in our case can be explained by the much larger number of genetic variants used but also by fewer clinical factors in our model. Indeed, it is often observed that adding a predictor to a model having a high AUC improves it by an amount smaller than what could be achieved by adding the same factor to a poorer model.

Furthermore, stratifying individuals by PRS quantiles revealed an association with a distinctive risk of severe COVID-19 in resulting groups. The highest PRS categories exhibited higher (adjusted OR up to 2.9 for the top 10% PRS) risk. This genetic basis for differences in disease severity among individuals also extended to the occurrence of mortality due to COVID-19 (adjusted OR = 4.3 for the top 10% PRS). These results demonstrate that polygenic risks can be employed to stratify patients and assess their risk of severe disease and mortality related to COVID-19.

Additional survival analysis revealed that the highest risk categories as defined by PRS not only exhibited higher risk for COVID-19 severity and mortality but also experienced an earlier onset of increased risk compared to the mean- and low-risk categories. These findings provide insights into both the overall risk for severe COVID-19 and how the risk varies by age.

The application of PRS in clinical settings presents many challenges. However, recent research has demonstrated their potential utility in the realm of healthcare, spanning a wide array of diseases [40–42]. As an illustrative example, the extended Breast and Ovarian Analysis of Disease Incidence and Carrier Estimation Algorithm (BOADICEA) model is employed to assess the risks of breast and ovarian cancer, by integrating various factors including personal risk indicators, familial cancer history, mammographic density, an assortment of lifestyle and hormonal aspects, genetic screening for high- and moderate-risk genes, as well as polygenic scores [43]. The validation of this model in a Dutch prospective cohort, with regard to breast cancer risk prediction, has demonstrated that the incorporation of PRS enhances the discriminatory capability from 0.53 to 0.64, compared to a model based solely on age, which currently serves as the sole determinant for breast cancer screening recommendations in the Dutch population [44]. Our study findings have indicated that the application of PRS in the context of COVID-19 holds potential implications for stratifying individuals according to their genetic predisposition towards disease severity.

In addition, several studies have found the positive impact of informing patients about their increased polygenic risk on behavioural patterns. For example, research has shown that when people are aware of their increased polygenic risk for certain diseases, such as cardiovascular disease or diabetes, they are more likely to engage in healthier lifestyles, including regular exercise, a balanced diet, and regular health checkups [45]. These behavioural changes can have a significant impact on disease prevention and treatment, ultimately leading to improved health outcomes. By providing patients with a thorough understanding of their genetic risk profiles, healthcare providers can encourage proactive measures to reduce potential risks and promote long-term health and well-being.

A few limitations of our study should be noted. First, despite the multi-ethnic and global nature of the HGI Release 7 meta-analysis, the participants were mostly of Western European descent [9]. While polygenic scores that incorporate GWAS data from diverse ancestries in addition to the target population have shown relative improvement in predictive accuracy compared to methods that rely solely on GWAS data from a single ancestry source [46], incorporating more genetic data from non-Western European populations into these models is expected to enhance their predictive accuracy, particularly for individuals from underrepresented populations. Second, the lack of specific clinical data required to accurately match the severity criteria used by the HGI consortium may have led to some inaccuracy in the classification of outcome measures for some participants.

Our study has several strengths. First, we evaluated the performance of polygenic risk models in the Russian population, which is a valuable contribution to understanding the limitations of PRS applicability, particularly in light of the over-representation of Western European population data in existing models. Secondly, for complex traits, previous research has shown that even the strongest GWAS hits tend to have modest effect sizes on risk and that all the genome-wide significant hits in combination explain only a small fraction of the disease risk heritability [47]. Subsequent studies have largely resolved this initial mystery by demonstrating that most of the missing heritability is due to numerous small-effect common variants that are not significant at current sample sizes [48, 49]. In this work, we employed a wide range of common SNPs for PRS calculation, which is an important addition to previously published models for severe COVID-19 prediction, where PRS values were calculated using only the most significant SNPs. Finally, recent publications have proposed the use of LP-WGS in conjunction with genotype imputation as an alternative to genotyping arrays for trait mapping and polygenic score calculation [24]. In our study, we also demonstrated good model performance using PRS based on LP-WGS data.

## Supporting information

Nostaeva et al. supplementary material 1 Nostaeva et al. supplementary material

Nostaeva et al. supplementary material 2 Nostaeva et al. supplementary material

## Data Availability

Personal genetic and clinical data are under restrictions and are available through collaboration with the St. Petersburg State Health Care Institution ‘City Hospital No. 40, Kurortny District’ hospital. The summary statistics from the COVID-19 Host Genetics Initiative consortium (release 7) are available at https://www.covid19hg.org/results/r7/. Within four weeks of publication, the PRS model for severe COVID-19 and the relevant metadata will be available at https://www.pgscatalog.org.

## Supplementary material

The supplementary material for this article can be found at http://doi.org/10.1017/S0950268824001778.
