# Supplementary material for: Case–control association study between polygenic risk score and COVID-19 severity in a Russian population using low-pass genome sequencing
Source: Epidemiol Infect. 2024 Dec 26;153:e13. doi: 10.1017/S0950268824001778 (PMC11748017; doi:10.1017/S0950268824001778)
Supplement: Nostaeva et al. supplementary material 1 — Nostaeva et al. supplementary material [file S0950268824001778sup001.docx]

**Epidemiology and Infection**

**Case–control association study between polygenic risk score and COVID-19 severity in a Russian population using low-pass genome sequencing**

Arina V. Nostaeva, Valentin S. Shimansky, Svetlana V. Apalko, Ivan A. Kuznetsov, Natalya N. Sushentseva, Oleg S. Popov, Anna Y. Asinovskaya, Sergey V. Mosenko, Lennart C. Karssen, Andrey M. Sarana, Yurii S. Aulchenko, Sergey G. Shcherbak

**Supplementary Figures**

**Supplementary Figure S1: Study flow diagram of patients’ filtering.**

**
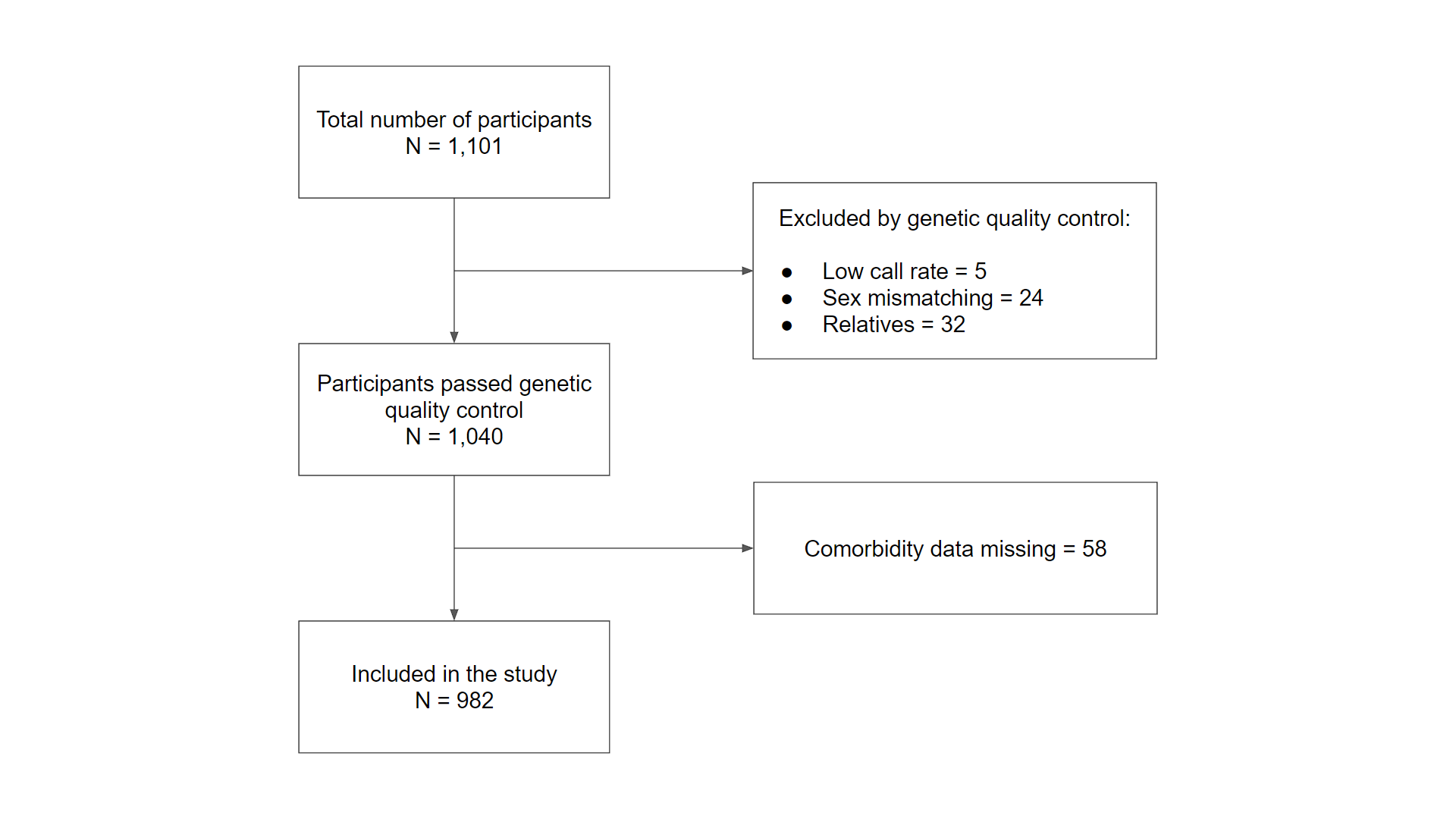
**

**Supplementary Figure S2: Association of PRS with COVID-19 severity.** All participants (N = 982) were stratified into three categories, based on their PRS: bottom decile, deciles 2–9, and top decile. The graph represents the partial effects of PRS on the COVID-19 severity with fixed values ​​of the covariates. Survival function was built using the multivariate Cox regression model adjusted by the covariates (sex, age, comorbidities, and the first 10 PCs). PRS values were varied as the mean PRS values ​​in each of the three groups described above.

**
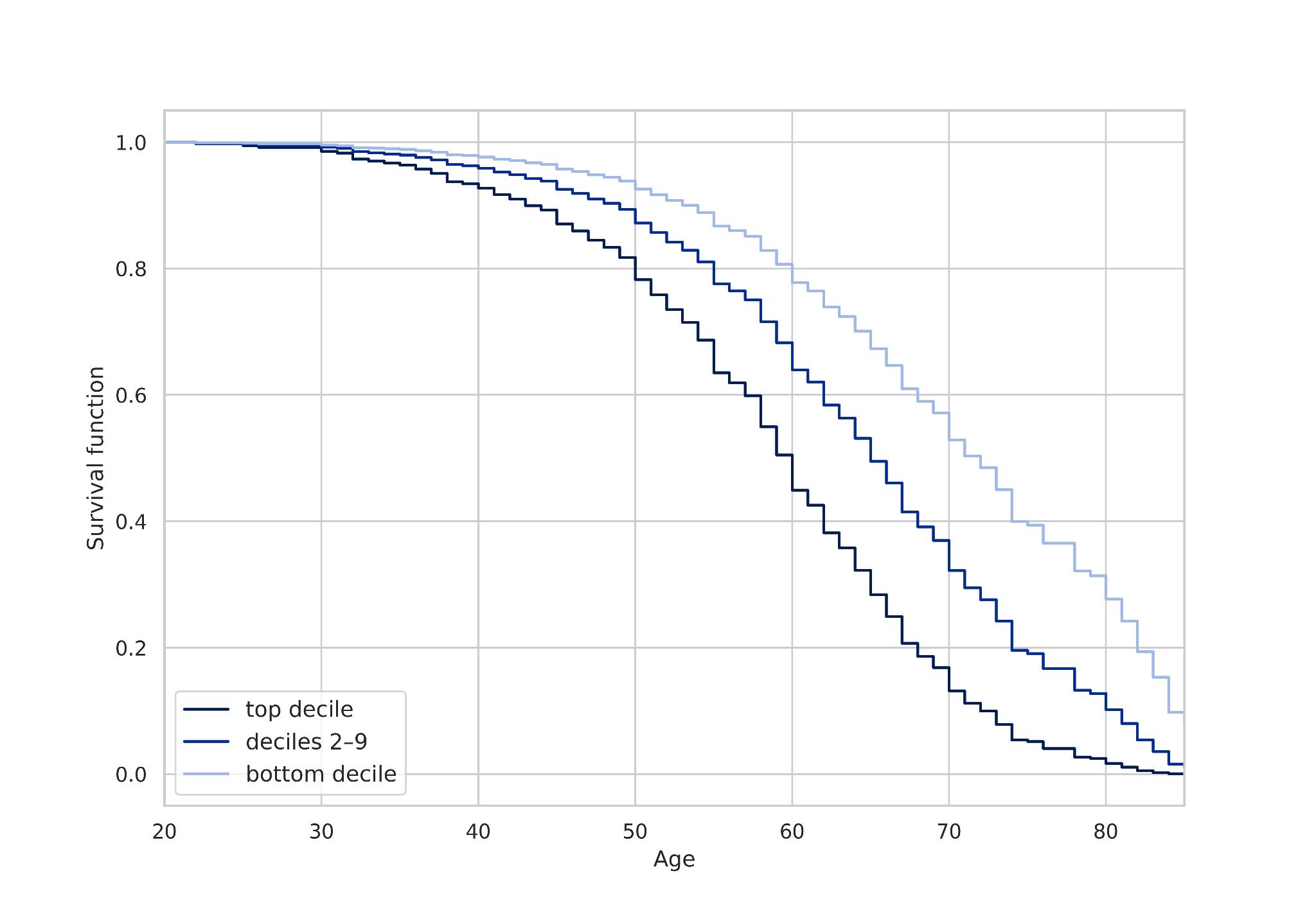
**

**Supplementary Figure S3: Association of PRS with COVID-19 mortality.** All participants (N = 982) were stratified into three categories, based on their PRS: bottom decile, deciles 2–9, and top decile. The Kaplan-Meier curve is plotted according to the PRS category.


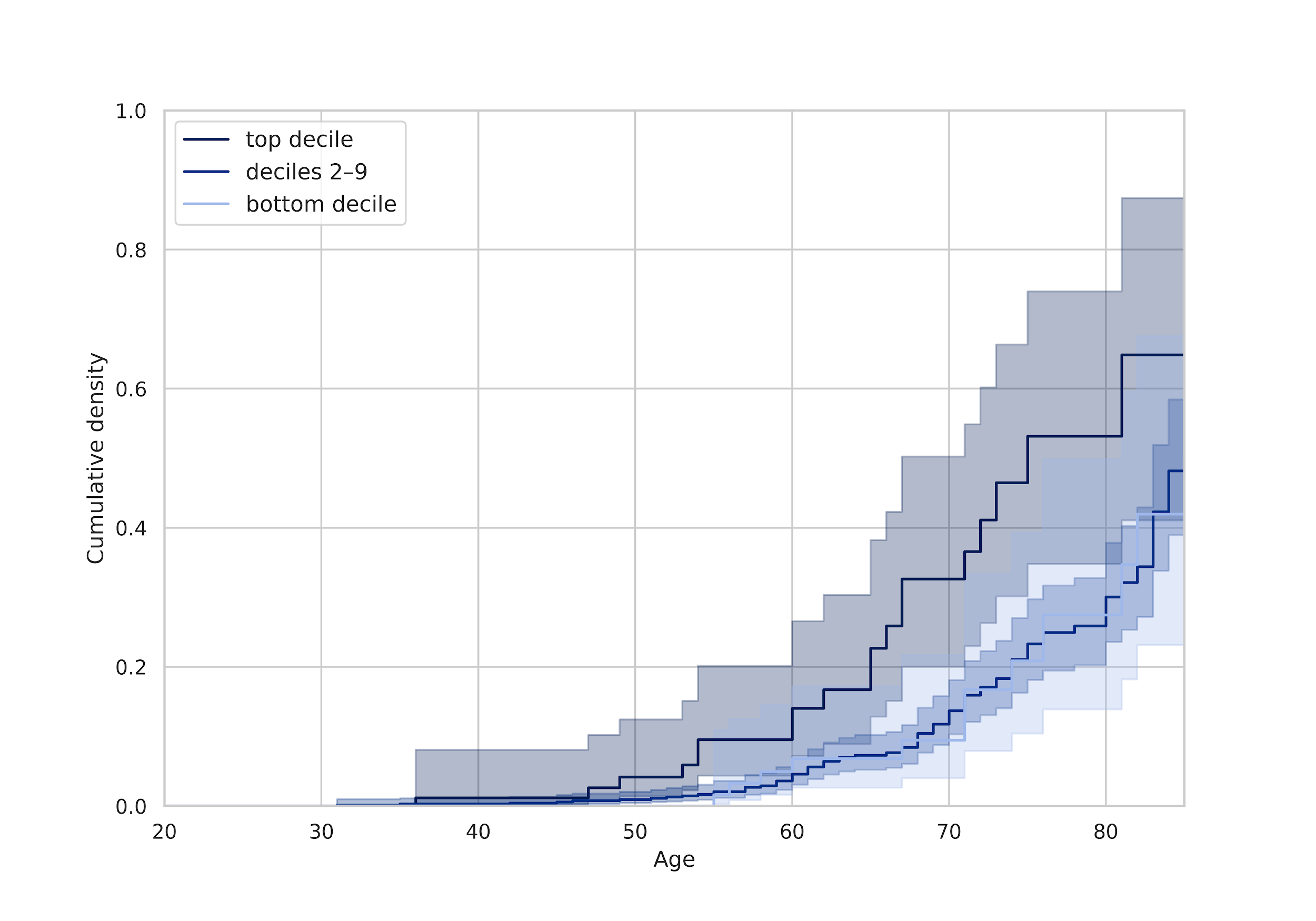


**Supplementary Figure S4: Association of PRS with COVID-19 mortality.** All participants (N = 982) were stratified into three categories, based on their PRS: bottom decile, deciles 2–9, and top decile. The graph represents the partial effects of PRS on the COVID-19 mortality with fixed values ​​of the covariates. Survival function was built using the multivariate Cox regression model adjusted by the covariates (sex, age, comorbidities, and the first 10 PCs). PRS values were varied as the mean PRS values ​​in each of the three groups described above.

**
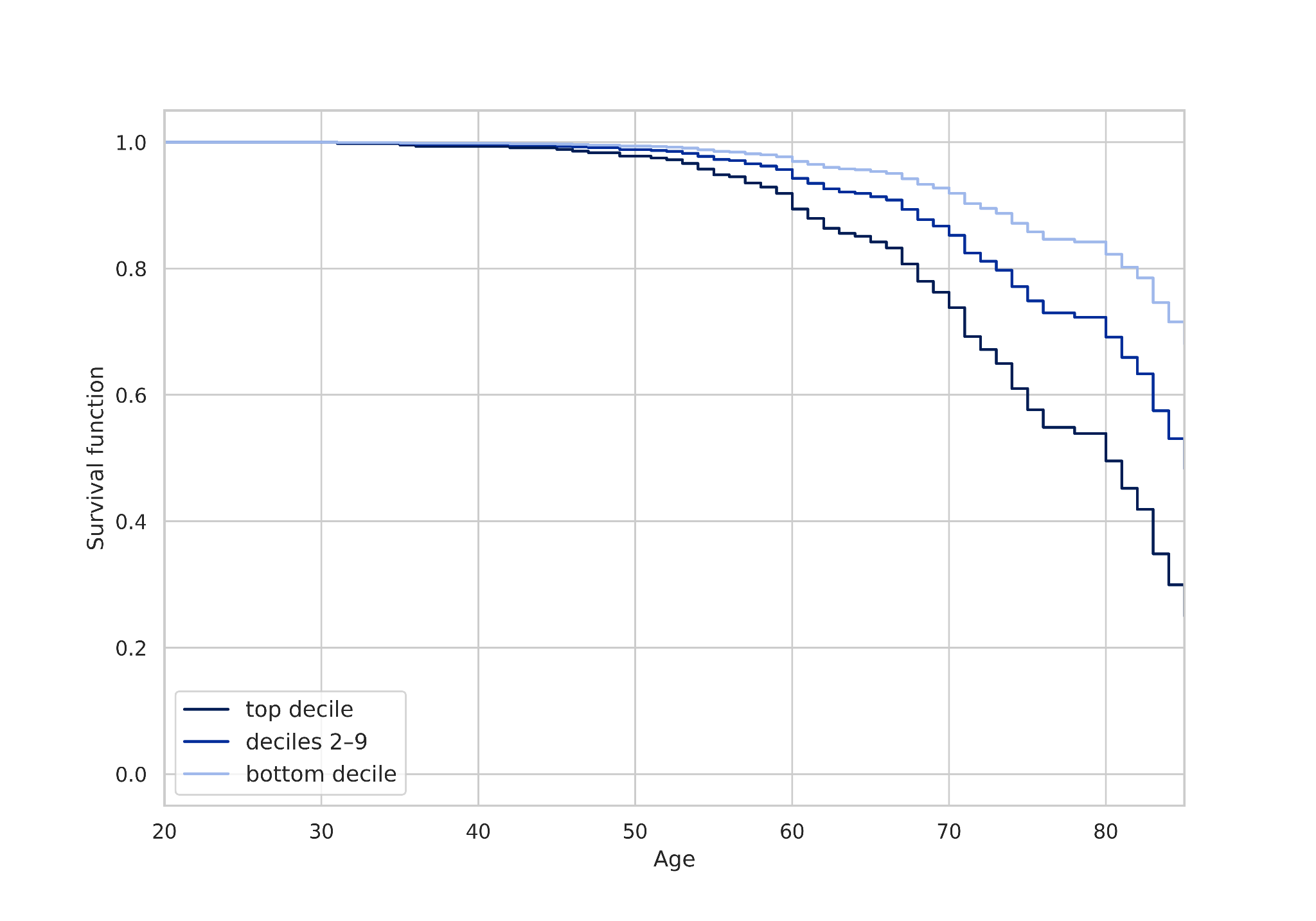
**
