# Supplementary material for: Case–control association study between polygenic risk score and COVID-19 severity in a Russian population using low-pass genome sequencing
Source: Epidemiol Infect. 2024 Dec 26;153:e13. doi: 10.1017/S0950268824001778 (PMC11748017; doi:10.1017/S0950268824001778)
Supplement: Nostaeva et al. supplementary material 2 — Nostaeva et al. supplementary material [file S0950268824001778sup002.docx]

**Epidemiology and Infection**

**Case–control association study between polygenic risk score and COVID-19 severity in a Russian population using low-pass genome sequencing**

Arina V. Nostaeva, Valentin S. Shimansky, Svetlana V. Apalko, Ivan A. Kuznetsov, Natalya N. Sushentseva, Oleg S. Popov, Anna Y. Asinovskaya, Sergey V. Mosenko, Lennart C. Karssen, Andrey M. Sarana, Yurii S. Aulchenko, Sergey G. Shcherbak

**Supplementary Tables**

**Supplementary Table 1:** Description of the distribution of myocardial infarction events in relation to COVID-19 severity and mortality outcomes.

| Myocardial  infarction | | COVID-severity | | | | COVID-mortality | | | |
| --- | --- | --- | --- | --- | --- | --- | --- | --- | --- |
|  |  | Cases | | Controls | | Cases | | Controls | |
|  |  | N | Mean age | N | Mean age | N | Mean age | N | Mean age |
| Comorbidity status | No | 275 | 61 | 584 | 54 | 102 | 68 | 757 | 54 |
|  | Yes | 44 | 73 | 79 | 69 | 31 | 76 | 92 | 69 |

**Supplementary Table 2:** Description of the distribution of heart failure events in relation to COVID-19 severity and mortality outcomes.

| Heart failure | | COVID-severity | | | | COVID-mortality | | | |
| --- | --- | --- | --- | --- | --- | --- | --- | --- | --- |
|  |  | Cases | | Controls | | Cases | | Controls | |
|  |  | N | Mean age | N | Mean age | N | Mean age | N | Mean age |
| Comorbidity status | No | 174 | 55 | 491 | 51 | 105 | 56 | 637 | 52 |
|  | Yes | 145 | 71 | 172 | 68 | 28 | 73 | 212 | 68 |

**Supplementary Table 3:** Description of the distribution of peripheral artery occlusive disease events in relation to COVID-19 severity and mortality outcomes.

| Peripheral artery  occlusive disease | | COVID-severity | | | | COVID-mortality | | | |
| --- | --- | --- | --- | --- | --- | --- | --- | --- | --- |
|  |  | Cases | | Controls | | Cases | | Controls | |
|  |  | N | Mean age | N | Mean age | N | Mean age | N | Mean age |
| Comorbidity status | No | 260 | 61 | 553 | 53 | 90 | 68 | 723 | 54 |
|  | Yes | 59 | 71 | 110 | 67 | 43 | 73 | 126 | 67 |

**Supplementary Table 4:** Description of the distribution of cerebrovascular disease events in relation to COVID-19 severity and mortality outcomes.

| Cerebrovascular  disease | | COVID-severity | | | | COVID-mortality | | | |
| --- | --- | --- | --- | --- | --- | --- | --- | --- | --- |
|  |  | Cases | | Controls | | Cases | | Controls | |
|  |  | N | Mean age | N | Mean age | N | Mean age | N | Mean age |
| Comorbidity status | No | 169 | 55 | 489 | 51 | 100 | 57 | 625 | 52 |
|  | Yes | 150 | 72 | 174 | 69 | 33 | 74 | 224 | 68 |

**Supplementary Table 5:** Description of the distribution of chronic obstructive pulmonary disease events in relation to COVID-19 severity and mortality outcomes.

| Chronic obstructive  pulmonary disease | | COVID-severity | | | | COVID-mortality | | | |
| --- | --- | --- | --- | --- | --- | --- | --- | --- | --- |
|  |  | Cases | | Controls | | Cases | | Controls | |
|  |  | N | Mean age | N | Mean age | N | Mean age | N | Mean age |
| Comorbidity status | No | 283 | 62 | 615 | 55 | 110 | 70 | 788 | 55 |
|  | Yes | 36 | 68 | 48 | 66 | 23 | 70 | 61 | 65 |

**Supplementary Table 6:** Description of the distribution of diabetes events in relation to COVID-19 severity and mortality outcomes.

| Diabetes | | COVID-severity | | | | COVID-mortality | | | |
| --- | --- | --- | --- | --- | --- | --- | --- | --- | --- |
|  |  | Cases | | Controls | | Cases | | Controls | |
|  |  | N | Mean age | N | Mean age | N | Mean age | N | Mean age |
| Comorbidity status | No | 246 | 61 | 595 | 54 | 94 | 68 | 747 | 55 |
|  | Yes | 73 | 67 | 68 | 66 | 39 | 73 | 102 | 64 |

**Supplementary Table 7:** Description of the distribution of kidney damage events in relation to COVID-19 severity and mortality outcomes.

| Kidney damage | | COVID-severity | | | | COVID-mortality | | | |
| --- | --- | --- | --- | --- | --- | --- | --- | --- | --- |
|  |  | Cases | | Controls | | Cases | | Controls | |
|  |  | N | Mean age | N | Mean age | N | Mean age | N | Mean age |
| Comorbidity status | No | 264 | 61 | 624 | 55 | 89 | 69 | 799 | 55 |
|  | Yes | 55 | 71 | 39 | 70 | 44 | 72 | 50 | 70 |

**Supplementary Table 8:** Logistic regression results for the full model with COVID-19 severity as an outcome and PRS as a continuous variable.

|  | coef | std err | z | P>\|z\| | [0.025 | 0.975] |
| --- | --- | --- | --- | --- | --- | --- |
| intercept | -1.528 | 0.148 | -10.338 | 4.75E-25 | -1.818 | -1.238 |
| age | 0.2727 | 0.104 | 2.618 | 8.85E-03 | 0.069 | 0.477 |
| sex | 0.5924 | 0.156 | 3.8 | 1.45E-04 | 0.287 | 0.898 |
| PRS | 0.4359 | 0.078 | 5.572 | 2.52E-08 | 0.283 | 0.589 |
| Myocardial infarction | -0.4745 | 0.245 | -1.939 | 5.25E-02 | -0.954 | 0.005 |
| Heart failure | 0.5087 | 0.209 | 2.439 | 1.47E-02 | 0.1 | 0.917 |
| Peripheral artery occlusive disease | -0.8205 | 0.237 | -3.461 | 5.39E-04 | -1.285 | -0.356 |
| Cerebrovascular disease | 0.534 | 0.201 | 2.661 | 7.80E-03 | 0.141 | 0.927 |
| Chronic obstructive pulmonary disease | 0.1994 | 0.256 | 0.778 | 4.37E-01 | -0.303 | 0.702 |
| Diabetes | 0.6888 | 0.209 | 3.295 | 9.84E-04 | 0.279 | 1.098 |
| Kidney damage | 0.7153 | 0.257 | 2.787 | 5.32E-03 | 0.212 | 1.218 |
| PC1 | 0.0283 | 0.098 | 0.289 | 7.73E-01 | -0.164 | 0.22 |
| PC2 | 0.1347 | 1.268 | 0.106 | 9.15E-01 | -2.35 | 2.619 |
| PC3 | -0.4851 | 1.264 | -0.384 | 7.01E-01 | -2.963 | 1.992 |
| PC4 | 0.208 | 0.575 | 0.361 | 7.18E-01 | -0.92 | 1.336 |
| PC5 | 0.2596 | 0.412 | 0.63 | 5.29E-01 | -0.548 | 1.067 |
| PC6 | 0.2689 | 0.272 | 0.987 | 3.24E-01 | -0.265 | 0.803 |
| PC7 | -0.0691 | 0.086 | -0.803 | 4.22E-01 | -0.238 | 0.1 |
| PC8 | -0.0737 | 0.073 | -1.004 | 3.15E-01 | -0.218 | 0.07 |
| PC9 | 0.0865 | 0.09 | 0.959 | 3.37E-01 | -0.09 | 0.263 |
| PC10 | 0.0432 | 0.077 | 0.562 | 5.74E-01 | -0.107 | 0.194 |

*- coef: the coefficient of the variable (log[odd ratio]);

- std err: the standard error of the coefficient;

- Z: the z value for the estimated coefficient and the standard error;

- P>|z|: the p-value associated with the value in the z value column;

- [0.025, 0.975]: 95% confident interval (CI) for the coefficient.

**Supplementary Table 9:** Logistic regression results for the full model with COVID-19 severity as an outcome and PRS as a binary variable.

|  | coef | std err | z | P>\|z\| | [0.025 | 0.975] |
| --- | --- | --- | --- | --- | --- | --- |
| intercept | -1.5995 | 0.15 | -10.652 | 1.71E-26 | -1.894 | -1.305 |
| 10% with the highest PRS values | 1.0795 | 0.234 | 4.604 | 4.14E-06 | 0.62 | 1.539 |
| age | 0.2693 | 0.103 | 2.607 | 9.13E-03 | 0.067 | 0.472 |
| sex | 0.5403 | 0.154 | 3.498 | 4.69E-04 | 0.238 | 0.843 |
| Myocardial infarction | -0.439 | 0.243 | -1.808 | 7.06E-02 | -0.915 | 0.037 |
| Heart failure | 0.5703 | 0.209 | 2.732 | 6.30E-03 | 0.161 | 0.979 |
| Peripheral artery occlusive disease | -0.8748 | 0.236 | -3.702 | 2.14E-04 | -1.338 | -0.412 |
| Cerebrovascular disease | 0.5203 | 0.2 | 2.6 | 9.32E-03 | 0.128 | 0.913 |
| Chronic obstructive pulmonary disease | 0.2688 | 0.255 | 1.053 | 2.92E-01 | -0.232 | 0.769 |
| Diabetes | 0.6799 | 0.209 | 3.26 | 1.11E-03 | 0.271 | 1.089 |
| Kidney damage | 0.689 | 0.255 | 2.697 | 6.99E-03 | 0.188 | 1.19 |
| PC1 | 0.0189 | 0.097 | 0.195 | 8.45E-01 | -0.171 | 0.209 |
| PC2 | 0.5335 | 1.262 | 0.423 | 6.73E-01 | -1.941 | 3.008 |
| PC3 | -0.8582 | 1.255 | -0.684 | 4.94E-01 | -3.318 | 1.602 |
| PC4 | 0.2265 | 0.534 | 0.424 | 6.72E-01 | -0.821 | 1.274 |
| PC5 | 0.266 | 0.385 | 0.691 | 4.90E-01 | -0.489 | 1.021 |
| PC6 | 0.2517 | 0.272 | 0.926 | 3.54E-01 | -0.281 | 0.784 |
| PC7 | -0.0536 | 0.087 | -0.619 | 5.36E-01 | -0.223 | 0.116 |
| PC8 | -0.0635 | 0.073 | -0.874 | 3.82E-01 | -0.206 | 0.079 |
| PC9 | 0.0825 | 0.09 | 0.92 | 3.58E-01 | -0.093 | 0.258 |
| PC10 | 0.0402 | 0.076 | 0.527 | 5.98E-01 | -0.109 | 0.19 |

**Supplementary Table 10:** Logistic regression results for the covariates-only model with COVID-19 severity as an outcome and PRS as a continuous variable.

|  | coef | std err | z | P>\|z\| | [0.025 | 0.975] |
| --- | --- | --- | --- | --- | --- | --- |
| intercept | -1.4475 | 0.142 | -10.183 | 2.37E-24 | -1.726 | -1.169 |
| age | 0.2555 | 0.101 | 2.518 | 1.18E-02 | 0.057 | 0.454 |
| sex | 0.5502 | 0.153 | 3.601 | 3.17E-04 | 0.251 | 0.85 |
| Myocardial infarction | -0.4594 | 0.24 | -1.911 | 5.60E-02 | -0.931 | 0.012 |
| Heart failure | 0.5499 | 0.206 | 2.671 | 7.56E-03 | 0.146 | 0.953 |
| Peripheral artery occlusive disease | -0.8703 | 0.234 | -3.72 | 1.99E-04 | -1.329 | -0.412 |
| Cerebrovascular disease | 0.4791 | 0.197 | 2.427 | 1.52E-02 | 0.092 | 0.866 |
| Chronic obstructive pulmonary disease | 0.2227 | 0.254 | 0.878 | 3.80E-01 | -0.274 | 0.72 |
| Diabetes | 0.6505 | 0.206 | 3.152 | 1.62E-03 | 0.246 | 1.055 |
| Kidney damage | 0.7441 | 0.253 | 2.936 | 3.33E-03 | 0.247 | 1.241 |
| PC1 | 0.0041 | 0.096 | 0.042 | 9.66E-01 | -0.184 | 0.192 |
| PC2 | 0.8017 | 1.253 | 0.64 | 5.22E-01 | -1.654 | 3.257 |
| PC3 | -1.0564 | 1.244 | -0.849 | 3.96E-01 | -3.494 | 1.381 |
| PC4 | 0.1782 | 0.456 | 0.391 | 6.96E-01 | -0.715 | 1.071 |
| PC5 | 0.2402 | 0.33 | 0.727 | 4.67E-01 | -0.407 | 0.887 |
| PC6 | 0.2175 | 0.232 | 0.939 | 3.48E-01 | -0.237 | 0.671 |
| PC7 | -0.0609 | 0.09 | -0.674 | 5.00E-01 | -0.238 | 0.116 |
| PC8 | -0.0629 | 0.072 | -0.869 | 3.85E-01 | -0.205 | 0.079 |
| PC9 | 0.1129 | 0.088 | 1.277 | 2.02E-01 | -0.06 | 0.286 |
| PC10 | 0.0453 | 0.075 | 0.602 | 5.47E-01 | -0.102 | 0.193 |

**Supplementary Table 11:** Logistic regression results for the PRS-only model with COVID-19 severity as an outcome and PRS as a continuous variable.

|  | coef | std err | z | P>\|z\| | [0.025 | 0.975] |
| --- | --- | --- | --- | --- | --- | --- |
| intercept | -0.7907 | 0.078 | -10.157 | 3.08E-24 | -0.943 | -0.638 |
| PRS | 0.3891 | 0.073 | 5.341 | 9.25E-08 | 0.246 | 0.532 |
| PC1 | -0.0102 | 0.092 | -0.111 | 9.12E-01 | -0.191 | 0.171 |
| PC2 | 0.4148 | 1.171 | 0.354 | 7.23E-01 | -1.88 | 2.709 |
| PC3 | -0.7998 | 1.176 | -0.68 | 4.97E-01 | -3.105 | 1.506 |
| PC4 | 0.1304 | 0.468 | 0.278 | 7.81E-01 | -0.788 | 1.048 |
| PC5 | 0.2196 | 0.336 | 0.653 | 5.14E-01 | -0.439 | 0.879 |
| PC6 | 0.307 | 0.296 | 1.037 | 3.00E-01 | -0.273 | 0.887 |
| PC7 | -0.0764 | 0.086 | -0.885 | 3.76E-01 | -0.246 | 0.093 |
| PC8 | -0.0735 | 0.078 | -0.947 | 3.43E-01 | -0.225 | 0.079 |
| PC9 | 0.0843 | 0.086 | 0.979 | 3.28E-01 | -0.085 | 0.253 |
| PC10 | -0.0401 | 0.072 | -0.555 | 5.79E-01 | -0.182 | 0.101 |

**Supplementary Table 12:** Logistic regression results for the full model with COVID-19 mortality as an outcome and PRS as a continuous variable.

|  | coef | std err | z | P>\|z\| | [0.025 | 0.975] |
| --- | --- | --- | --- | --- | --- | --- |
| intercept | -4.0733 | 0.301 | -13.552 | 7.67E-42 | -4.662 | -3.484 |
| age | 0.3928 | 0.173 | 2.276 | 2.28E-02 | 0.055 | 0.731 |
| sex | 0.6529 | 0.241 | 2.714 | 6.65E-03 | 0.181 | 1.124 |
| PRS | 0.4102 | 0.12 | 3.407 | 6.58E-04 | 0.174 | 0.646 |
| Myocardial infarction | -0.2595 | 0.291 | -0.892 | 3.72E-01 | -0.83 | 0.311 |
| Heart failure | 1.6768 | 0.295 | 5.693 | 1.25E-08 | 1.1 | 2.254 |
| Peripheral artery occlusive disease | -0.7706 | 0.28 | -2.755 | 5.87E-03 | -1.319 | -0.222 |
| Cerebrovascular disease | 0.9699 | 0.284 | 3.416 | 6.36E-04 | 0.413 | 1.526 |
| Chronic obstructive pulmonary disease | 0.6209 | 0.316 | 1.964 | 4.96E-02 | 0.001 | 1.241 |
| Diabetes | 0.4064 | 0.269 | 1.508 | 1.31E-01 | -0.122 | 0.935 |
| Kidney damage | 1.2328 | 0.29 | 4.246 | 2.17E-05 | 0.664 | 1.802 |
| PC1 | 0.0185 | 0.145 | 0.128 | 8.98E-01 | -0.266 | 0.303 |
| PC2 | 0.8495 | 2.147 | 0.396 | 6.92E-01 | -3.358 | 5.057 |
| PC3 | -1.7958 | 2.017 | -0.89 | 3.73E-01 | -5.75 | 2.158 |
| PC4 | 1.0886 | 1.083 | 1.005 | 3.15E-01 | -1.034 | 3.211 |
| PC5 | 0.6319 | 0.786 | 0.804 | 4.22E-01 | -0.909 | 2.173 |
| PC6 | 0.3957 | 0.52 | 0.761 | 4.47E-01 | -0.623 | 1.415 |
| PC7 | -0.0428 | 0.11 | -0.39 | 6.97E-01 | -0.258 | 0.172 |
| PC8 | 0.0004 | 0.149 | 0.002 | 9.98E-01 | -0.292 | 0.293 |
| PC9 | -0.0769 | 0.164 | -0.47 | 6.38E-01 | -0.398 | 0.244 |
| PC10 | 0.037 | 0.118 | 0.313 | 7.54E-01 | -0.195 | 0.269 |

**Supplementary Table 13:** Logistic regression results for the full model with COVID-19 mortality as an outcome and PRS as a binary variable.

|  | coef | std err | z | P>\|z\| | [0.025 | 0.975] |
| --- | --- | --- | --- | --- | --- | --- |
| intercept | -4.2696 | 0.316 | -13.493 | 1.71E-41 | -4.89 | -3.649 |
| 10% with the highest PRS values | 1.4697 | 0.342 | 4.299 | 1.72E-05 | 0.8 | 2.14 |
| age | 0.3729 | 0.172 | 2.164 | 3.04E-02 | 0.035 | 0.711 |
| sex | 0.611 | 0.241 | 2.535 | 1.12E-02 | 0.139 | 1.083 |
| Myocardial infarction | -0.2145 | 0.292 | -0.736 | 4.62E-01 | -0.786 | 0.357 |
| Heart failure | 1.7802 | 0.304 | 5.854 | 4.79E-09 | 1.184 | 2.376 |
| Peripheral artery occlusive disease | -0.8416 | 0.281 | -2.991 | 2.78E-03 | -1.393 | -0.29 |
| Cerebrovascular disease | 1.014 | 0.292 | 3.478 | 5.06E-04 | 0.443 | 1.586 |
| Chronic obstructive pulmonary disease | 0.6964 | 0.319 | 2.185 | 2.89E-02 | 0.072 | 1.321 |
| Diabetes | 0.4112 | 0.27 | 1.525 | 1.27E-01 | -0.117 | 0.94 |
| Kidney damage | 1.2124 | 0.291 | 4.163 | 3.14E-05 | 0.642 | 1.783 |
| PC1 | 0.0295 | 0.145 | 0.204 | 8.39E-01 | -0.255 | 0.314 |
| PC2 | 1.2105 | 2.166 | 0.559 | 5.76E-01 | -3.035 | 5.457 |
| PC3 | -2.1391 | 2.04 | -1.049 | 2.94E-01 | -6.137 | 1.859 |
| PC4 | 1.092 | 1.076 | 1.015 | 3.10E-01 | -1.017 | 3.201 |
| PC5 | 0.6214 | 0.779 | 0.798 | 4.25E-01 | -0.906 | 2.148 |
| PC6 | 0.4605 | 0.529 | 0.87 | 3.84E-01 | -0.577 | 1.498 |
| PC7 | -0.0315 | 0.111 | -0.282 | 7.78E-01 | -0.25 | 0.187 |
| PC8 | 0.0104 | 0.157 | 0.066 | 9.47E-01 | -0.298 | 0.319 |
| PC9 | -0.0644 | 0.162 | -0.397 | 6.91E-01 | -0.382 | 0.254 |
| PC10 | 0.0446 | 0.118 | 0.379 | 7.04E-01 | -0.186 | 0.275 |

**Supplementary Table 14:** Multivariate Cox regression results for the model with COVID-19 severity as an outcome and PRS as a continuous variable.

|  | coef | Hazard ratio (HR) | HR lower 95% | HR upper 95% | p | -log2(p) |
| --- | --- | --- | --- | --- | --- | --- |
| sex | 0.74 | 2.1 | 1.66 | 2.66 | <0.005 | 30.72 |
| PRS | 0.33 | 1.39 | 1.24 | 1.57 | <0.005 | 24.71 |
| Myocardial infarction | -0.77 | 0.46 | 0.33 | 0.66 | <0.005 | 16 |
| Heart failure | -0.43 | 0.65 | 0.49 | 0.86 | <0.005 | 8.38 |
| Peripheral artery occlusive disease | -0.12 | 0.88 | 0.64 | 1.22 | 0.46 | 1.13 |
| Cerebrovascular disease | -0.49 | 0.61 | 0.47 | 0.8 | <0.005 | 11.57 |
| Chronic obstructive pulmonary disease | -0.21 | 0.81 | 0.57 | 1.16 | 0.25 | 1.99 |
| Diabetes | 0.14 | 1.15 | 0.87 | 1.52 | 0.32 | 1.64 |
| Kidney damage | 0.04 | 1.04 | 0.75 | 1.45 | 0.8 | 0.32 |
| PC1 | 0.01 | 1.01 | 0.88 | 1.17 | 0.85 | 0.23 |
| PC2 | 0.2 | 1.22 | 0.16 | 9.33 | 0.85 | 0.24 |
| PC3 | -0.45 | 0.64 | 0.09 | 4.55 | 0.65 | 0.62 |
| PC4 | 0.59 | 1.8 | 0.68 | 4.79 | 0.24 | 2.07 |
| PC5 | 0.45 | 1.57 | 0.77 | 3.2 | 0.21 | 2.25 |
| PC6 | 0.22 | 1.25 | 0.78 | 1.99 | 0.35 | 1.5 |
| PC7 | -0.01 | 0.99 | 0.86 | 1.13 | 0.84 | 0.25 |
| PC8 | -0.05 | 0.95 | 0.85 | 1.06 | 0.36 | 1.48 |
| PC9 | 0.13 | 1.14 | 0.99 | 1.31 | 0.07 | 3.82 |
| PC10 | 0.04 | 1.04 | 0.93 | 1.17 | 0.49 | 1.04 |

**Supplementary Table 15:** Multivariate Cox regression results for the model with COVID-19 mortality as an outcome and PRS as a continuous variable.

|  | coef | Hazard ratio (HR) | HR lower 95% | HR upper 95% | p | -log2(p) |
| --- | --- | --- | --- | --- | --- | --- |
| sex | 0.9 | 2.47 | 1.68 | 3.63 | <0.005 | 17.75 |
| PRS | 0.37 | 1.44 | 1.19 | 1.75 | <0.005 | 12.29 |
| Myocardial infarction | -0.61 | 0.55 | 0.35 | 0.85 | 0.01 | 7 |
| Heart failure | 0.55 | 1.73 | 1.05 | 2.85 | 0.03 | 4.92 |
| Peripheral artery occlusive disease | -0.04 | 0.96 | 0.64 | 1.46 | 0.86 | 0.22 |
| Cerebrovascular disease | -0.01 | 0.99 | 0.63 | 1.57 | 0.98 | 0.03 |
| Chronic obstructive pulmonary disease | 0.2 | 1.22 | 0.76 | 1.97 | 0.42 | 1.26 |
| Diabetes | 0.12 | 1.13 | 0.75 | 1.7 | 0.55 | 0.86 |
| Kidney damage | 0.34 | 1.41 | 0.93 | 2.12 | 0.1 | 3.3 |
| PC1 | 0.01 | 1.01 | 0.8 | 1.26 | 0.96 | 0.05 |
| PC2 | -0.21 | 0.81 | 0.03 | 21.2 | 0.9 | 0.15 |
| PC3 | -0.24 | 0.78 | 0.04 | 15.96 | 0.87 | 0.19 |
| PC4 | 0.44 | 1.55 | 0.28 | 8.74 | 0.62 | 0.69 |
| PC5 | 0.22 | 1.24 | 0.34 | 4.54 | 0.74 | 0.43 |
| PC6 | 0.28 | 1.33 | 0.6 | 2.96 | 0.49 | 1.03 |
| PC7 | 0 | 1 | 0.84 | 1.2 | 0.98 | 0.02 |
| PC8 | -0.04 | 0.96 | 0.77 | 1.2 | 0.71 | 0.5 |
| PC9 | -0.01 | 0.99 | 0.76 | 1.29 | 0.97 | 0.05 |
| PC10 | 0.04 | 1.04 | 0.86 | 1.25 | 0.72 | 0.48 |
